# Structure-Guided Immobilization of an Evolved Unspecific Peroxygenase

**DOI:** 10.3390/ijms20071627

**Published:** 2019-04-02

**Authors:** Patricia Molina-Espeja, Paloma Santos-Moriano, Eva García-Ruiz, Antonio Ballesteros, Francisco J. Plou, Miguel Alcalde

**Affiliations:** 1 Department of Biocatalysis, Institute of Catalysis, CSIC, Cantoblanco, 28049 Madrid, Spain; patricia.molina@icp.csic.es (P.M.-E.); palomacarmen.santos@universidadeuropea.es (P.S.-M.); eva.garcia.ruiz@csic.es (E.G.-R.); a.ballesteros@icp.csic.es (A.B.); 2 Applied Biotechnology Group, Faculty of Biomedical and Health Sciences, Universidad Europea de Madrid, Urbanización El Bosque, Villaviciosa de Odón, 28670 Madrid, Spain

**Keywords:** directed evolution, structure-guided immobilization, oxyfunctionalization, unspecific peroxygenase

## Abstract

Unspecific peroxygenases (UPOs) are highly promiscuous biocatalyst with self-sufficient mono(per)oxygenase activity. A laboratory-evolved UPO secreted by yeast was covalently immobilized in activated carriers through one-point attachment. In order to maintain the desired orientation without compromising the enzyme’s activity, the S221C mutation was introduced at the surface of the enzyme, enabling a single disulfide bridge to be established between the support and the protein. Fluorescence confocal microscopy demonstrated the homogeneous distribution of the enzyme, regardless of the chemical nature of the carrier. This immobilized biocatalyst was characterized biochemically opening an exciting avenue for research into applied synthetic chemistry.

## 1. Introduction

The selective oxyfunctionalization of organic molecules is fundamental for the synthesis of chemicals, building blocks, specialized and functionalized polymers, as well as pharmacological compounds. Most of these industrial transformations take place under aggressive and non-environmentally friendly conditions [1,2]. For example, the cumene process for the production of phenol from benzene is energetically unbalanced, requiring the use of high temperature and pressure (over 200 °C and 30 atmospheres), and it is associated with the release of poisoned metallic catalysts and toxic by-products. However, the use of highly selective enzymes to introduce oxygen functionalities as an alternative to chemical catalysis may overcome these hurdles, allowing these processes to be carried out in water at room temperature and under atmospheric pressure, and releasing very few side-products [3,4,5]. Among oxyfunctionalization biocatalysts, P450 monooxygenases have been exhaustively studied and engineered during the last few decades in order to meet with industrial standards. Unfortunately, P450s are general membrane-associated, labile and require expensive redox cofactors (NAD(P)H) and auxiliary flavoproteins, precluding their implementation in many technological settings.

Almost 15 years ago, the first unspecific peroxygenase (UPO, EC 1.11.2.1) was reported [6], a heme-thiolate enzyme considered by many as the missing link between heme peroxidases and P450s in terms of their catalytic mechanism. However, unlike the latter, UPO is very stable and soluble, working as a self-sufficient mono(per)oxygenase fueled by catalytic concentrations of H_2_O_2_. UPOs can act on a wide variety of compounds, producing the hydroxylation of aliphatic compounds, aromatic and heterocyclic oxygenation, epoxidation of alkenes, dealkylation reactions and more [7]. Hence, UPOs has been studied for the preparation of active pharmaceuticals, for example in the synthesis of the pain killer paracetamol and 4′-hydroxydiclofenac, or to reassemble the activity of human liver P450s during the production of toxicants and carcinogens. Likewise, it has been used to generate agrochemicals (including several pesticide precursors), in the cosmetic and food sectors, and also for the bioremediation of xenobiotics (including recalcitrant polyaromatic hydrocarbons -PAHs) [7,8,9].

Yet, there are three premises that must be fulfilled to make UPO a practical industrial biocatalyst: (i) a protein engineering platform with which to adapt the enzyme’s attributes to the specific industrial requirements -in terms of stability and substrate scope-; (ii) protocols to over-express variants of this enzyme in suitable heterologous hosts; and (iii) the immobilization of the enzyme to inert carriers that favor enzyme reusability, and the rapid separation of catalysts and reactants.

We have resolved the first need by subjecting the UPO from the edible mushroom *Agrocybe aegerita* (*Aae*UPO) to five rounds of directed evolution for functional expression in *Saccharomyces cerevisiae* [10]. For this purpose, we combined different library creation methods including random mutagenesis with in vivo DNA shuffling, as well as focused mutagenesis at the signal peptide to give rise to a readily secreted UPO variant (named PaDa-I). Along this directed evolution campaign, four mutations were introduced in the secretion leader with five more accumulated in the mature protein. The PaDa-I variant showed similar properties to that of the homologously expressed wild type *Aae*UPO in terms of kinetic constants and activity pH profiles, whereas it displayed an increased stability and improved secretion in yeast. Additionally, PaDa-I was transferred to the methylotrophic yeast *Pichia pastoris* (*Komagataella phaffii*) (producing up to ~0.3 g UPO/L in a bioreactor), and thereby making a step forward towards the second premise [11].

Attempts have been made to address the third premise by immobilizing *Aae*UPO [12,13], but without achieving adequate control over the immobilized enzyme. Indeed, random interactions with activated supports typically produce heterogeneous populations with different carrier arrangements and enzyme orientations, which are not particularly reproducible [14,15,16,17]. When dealing with selective transformations, it is necessary to precisely orient the enzyme during immobilization, such as in the construction of nanobiodevices and for flow biocatalysis [18]. In these cases, controlled/oriented immobilization can be achieved by using antibodies, histidine tags, biotin/avidin systems or disulfide bonds [19]. The latter appears to be a particularly valuable approach, as demonstrated in the immobilization of a glucose-6-phosphate dehydrogenase via thiol-disulfide interchange [20], or through site-directed oriented immobilization of a genetically modified thermophilic lipase [21]. To date, the few reported immobilization protocols for UPOs are based on the wild-type *Aae*UPO, which precludes protein engineering work aimed at driving enzyme immobilization. Conversely, structure-guided immobilization could be achieved by using the evolved PaDa-I variant, which is easily produced in yeast and its derived variants can be ad hoc designed through further genetic engineering.

In the present study, we have explored such processes by immobilizing the evolved PaDa-I mutant harnessing an oriented-attachment strategy. Through structure-guided mutagenesis, a Cys residue was initially introduced at the surface of the enzyme, and this variant was immobilized by establishing unique S-S bonds on different activated supports. The biocatalysts were inspected by fluorescence confocal microscopy and characterized in terms of their immobilization yield, activity and stability in response to temperature, organic solvents and changes in pH.

## 2. Results and Discussion

### 2.1. Unspecific Peroxygenase (UPO) Mutant Engineering (PaDa-I-Cys, S221C)

**Figure 1:**
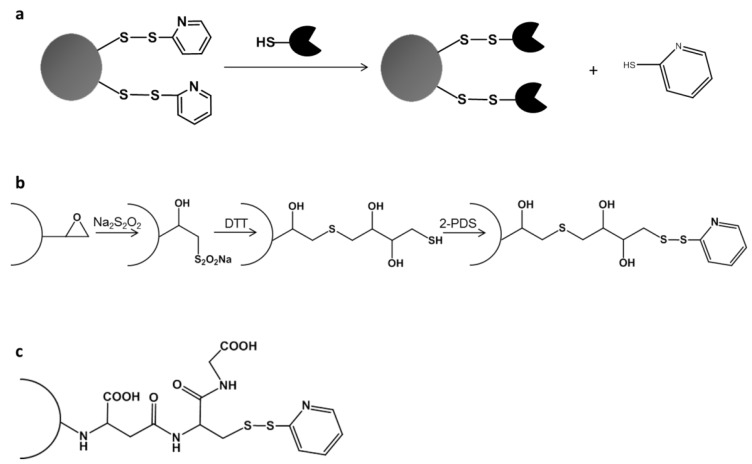
Directed unique-point covalent immobilization (DUCI) strategy: (**a**) general approach for oriented one-point immobilization; (**b**) chemical modification protocol for EC-EP203 (C-I) activation of the Sepabeads; (**c**) activated thiol-Sepharose (C-II). 2,2’-dithiodipyridine (2-PDS); 1,4-dithiothreitol (DTT).

We immobilized the PaDa-I mutant by the Directed Unique-point Covalent Immobilization (DUCI) strategy, as summarized in Figure 1. DUCI relies on the engineering of specific Cys-mutants, establishing selective covalent interactions between the enzyme and the support. Irrespective of the chemical nature of the activated support, one point site-directed Cys-mutants promote a specific attachment to the carrier through a single disulfide bridge in such a manner that the enzyme orientation can be controlled. Since our departure point was the evolved PaDa-I variant, its recently published crystal structure (Protein Data Bank (PDB) entry: 5OXU) [22] was carefully analyzed. This UPO variant is formed by a quite compact but complex polypeptide (328 amino acids excluding the 43 amino acid signal peptide), with a characteristic thiolate ligand axial to the heme prosthetic group, a Mg^2+^ binding site, a halide binding site and a funnel-shaped access channel to the substrate binding site formed by aromatic (mostly Phe) residues. It is worth noting that there are no free SH functional groups, and only one disulfide bridge between Cys278 and Cys319 serves to stabilize the C-terminal region of the protein.

**Figure 2:**
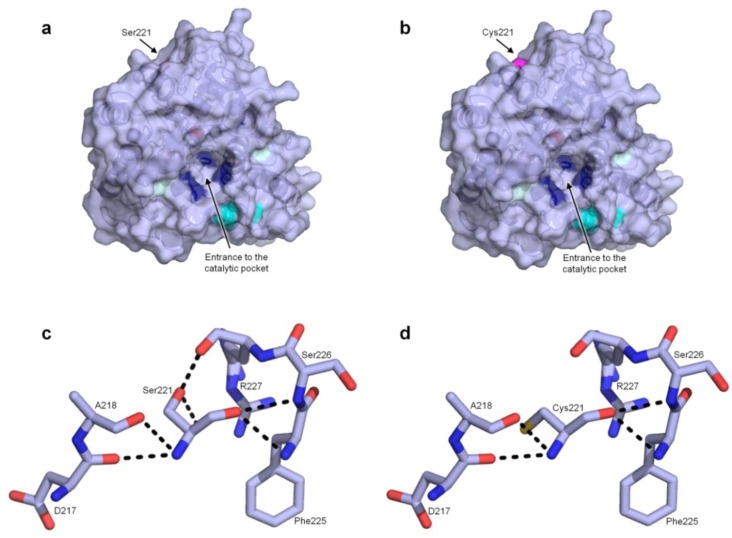
(**a**,**b**) general structure of PaDa-I/ PaDa-I-Cys with the relative position of the S221C marked. The V75I and I248V mutations are shown in light blue, whereas the V57A, L67F and F311L mutations are not shown, as they are situated at the back of the model. Cyan stands for C278-C319, forming the disulfide bridge, dark blue represents Phe residues in the heme channel orienting/accommodating the substrates while the heme iron is shown as a red sphere; (**c**) and (**d**) the S221C mutation and its interactions with the surrounding residues. Dark blue: nitrogen, red: oxygen, yellow: sulfur.

Ser residues appeared to be the best option to be replaced by Cys since they are quite abundant (18 residues), and they have a similar polarity and side-chain length to Cys. Accordingly, we examined the PaDa-I structure to find a suitable surface-exposed Ser residue, far from the catalytic regions and preferably, opposite to them. We also looked for residues that did not interfere with any of the 5 mutations introduced in the evolved mature PaDa-I (V57A-L67F-V75I-I248V-F311L) or with the existing Cys residues in order to avoid undesired disulfide bonds. After careful inspection, the best candidate was Ser221, located at the surface and in a corner of the protein structure, far from most of the catalytic determinants, Figure 2a,b. Using PaDa-I as starting point, the S221C mutant was generated (named PaDa-I-Cys), and it was functionally expressed in *S. cerevisiae*. To determine if any significant change of activities occurred because of the introduction of the S221C mutation, we assessed both the PaDa-I and the PaDa-I-Cys enzymes against two typical substrates, NBD (5-nitro-1,3-benzodioxole), a peroxygenative compound, and ABTS (2,2′-azino-bis(3-ethylbenzothiazoline-6-sulfonic acid)), a peroxidative compound, Table 1. While the peroxygenative:peroxidative activity ratio did not vary significantly, the secretion dropped by 40% for the PaDa-I-Cys variant.

As can be seen in Figure 2c, Ser221 interacts with the surrounding Asp217, Ala218, Phe225, Ser226 and Arg227 residues through H-bonds. According to our model, the interaction of the OH from the side chain of Ser221 with the oxygen atom of the main chain of Arg227 may be interrupted after mutation, Figure 2d. Besides, the additional polar contact between the OH from Ser221 and a molecule of water, seems to be removed when introducing the Cys221 mutation. These two interruptions might be the reason behind the noticeable drop in secretion.

### 2.2. Directed Unique-Point Covalent Immobilization (DUCI) Strategy

**Figure 3:**
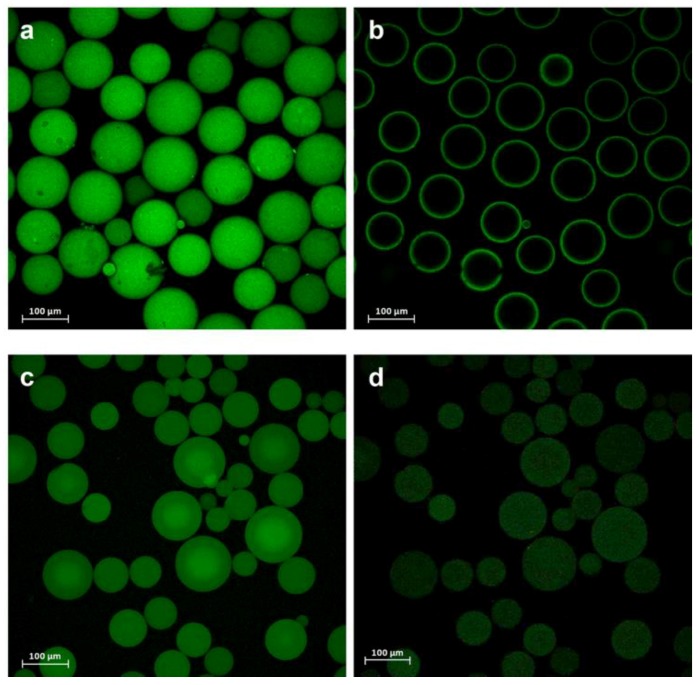
Confocal images reconstructing the fluorescein isothiocyanate (FITC)-labeled PaDa-I-Cys mutant (**a**) immobilized on C-I and (**c**) C-II. (**b**) Deep z-section scan of the C-I and (**d**) C-II biocatalysts.

The PaDa-I-Cys mutant was subjected to DUCI on two different commercial carriers: Sepabeads EC-EP203 (C-I), a polymethacrylate epoxy activated carrier, and thiol-Sepharose^®^ (C-II), an agarose carrier pre-activated with 2,2’-dithiodipyridine (2-PDS), Figure 1b,c. In this case, the C-I beads had also to be pre-activated by chemical modification with 2-PDS, following a modified protocol of that reported elsewhere [23] (see Methods for details). The immobilization yields (recovery of activity) for C-I and C-II were ~15%, both carriers producing 0.7 ABTS U g^−1^ of support. The controls performed with PaDa-I showed negligible activity, indicating that there was selective binding of the PaDa-I-Cys mutant through a single S-S bond and ruling out any other kind of interaction with the carriers (e.g., ionic or hydrophobic adsorption). To visualize the enzyme bound to the carriers, fluorescence confocal microscopy was performed with protein previously labelled with fluorescein isothiocyanate (FITC) [24,25], Figure 3.

As expected, whilst PaDa-I did not appear to attach to C-II, there was a small amount of PaDa-I bound to C-I, probably as a result of adsorption to the polymethacrylate or non-selective covalent immobilization to the epoxy groups not SH activated (data not shown). Although C-I had an average pore diameter of 130 nm, with an average pore volume of 1.19 cm^3^ g^−1^ and a specific surface area of 43 m^2^ g^−1^ [26], the PaDa-I-Cys was mainly immobilized on the surface of the carrier (Figure 3a,b), as also seen previously with an immobilized sterol esterase on similar carriers [24]. Unlike C-I, the higher swelling factor of C-II (4–5 mL per gram of gel) allowed the PaDa-I-Cys to diffuse through the pores, accessing the inner structure of the carrier, Figure 3c,d. Thus, despite the weaker fluorescence intensity for C-II, the total activity recovered was the same, indicating a similar amount of the PaDa-I-Cys mutant attached to both supports.

### 2.3. PaDa-I-Cys-DUCI Characterization

The PaDa-I-Cys (biocatalyst) immobilized on C-I and C-II was characterized biochemically, measuring the peroxygenative and peroxidative activities with NBD and ABTS, respectively. While the immobilized C-I showed no activity against NBD, C-II produced up to 0.240 (± 0.002) U g^−1^ of biocatalyst on NBD. By contrast, the activity of both C-I and C-II was similar on ABTS (0.740 ± 0.01 U g^−1^). Given that the same DUCI strategy was employed for both carriers, C-I may interfere with the NBD assay, impeding the reliable measurement of NBD activity. The activity following C-II immobilization was further assessed in two reactions of industrial interest by high-performance liquid chromatography–photodiode array (HPLC–PDA): the epoxidation of styrene to styrene oxide; and the hydroxylation of naphthalene to 1-naphthol. Activities of 0.066 (±0.005) and of 4.5 (±0.5) × 10^−3^ U g^−1^ were observed for styrene and naphthalene, respectively. Moreover, the selectivity of the reactions was maintained, showing only minor traces of side-products, consistent with the activity of the previously reported wild type *Aae*UPO [27,28].

**Figure 4:**
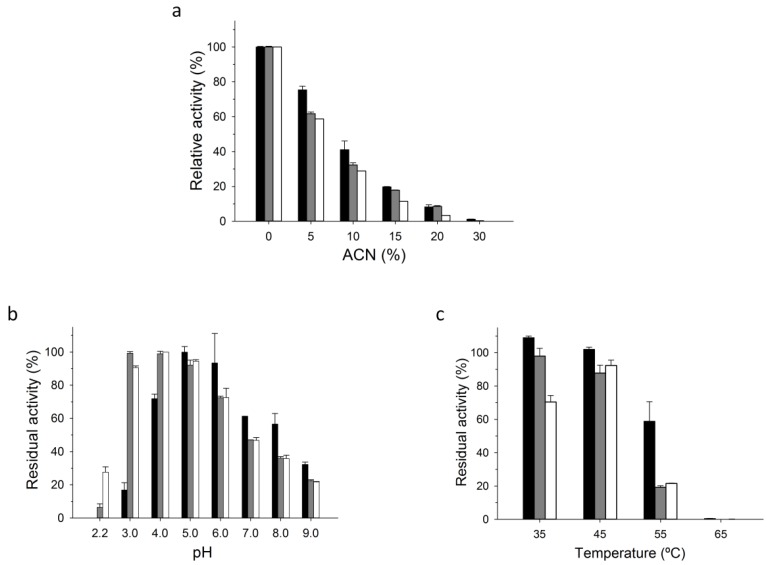
Biochemical characterization. (**a**) Activity in the presence of ACN; (**b**) pH stability and (**c**) thermostability after a 1 h incubation (the value for 100 % of activity was taken as the initial activity of each enzyme without incubation at room temperature, 25 °C). Activity was measured in sodium phosphate/citrate buffer (pH 4.4, 100 mM) containing ABTS (0.3 mM) and H_2_O_2_ (2 mM). Black bars, PaDa-I-Cys immobilized on C-II; grey bars, free PaDa-I-Cys; white bars, PaDa-I.

Further characterization was carried out in order to compare the immobilized PaDa-I-Cys variant on C-II, the free PaDa-I-Cys and the free PaDa-I variant, Figure 4. We did not include the wild-type *Aae*UPO in this set of experiments due to its equivalent performance to PaDa-I [10]. Since many of the transformations performed by UPO require the presence of acetonitrile (ACN) to help the solubilization of the reactants and products, we measured the enzyme’s activity in the presence of this organic solvent. A slightly higher relative activity was observed at low ACN concentrations for C-II than with the soluble mutant enzymes, Figure 4a. The stability against temperature and pH was also measured. Both PaDa-I-Cys and PaDa-I showed similar pH profiles, indicating that the S221C mutation did not negatively affected the pH stability of the enzyme, Figure 4b,c. The immobilized C-II was more stable at pH 5.0 and above, whereas its activity was diminished dramatically at pH 4.0 and below. With regard kinetic thermostability, the immobilized C-II retained ~3-fold more residual activity at 55 °C than its soluble counterparts.

We also tested the immobilized C-II to assess the leaching of the enzyme and its reuse capability. Up to 8 washes were carried out with the enzyme’s stability buffer (50 mM potassium phosphate buffer pH 7.0) and no activity was detected in the supernatant, showing that the PaDa-I-Cys was tightly bound to the support. Concerning operational stability in the batch, no reuse was possible. Unfortunately, UPO suffers of peroxide suicide inactivation [7], as all heme-containing peroxidases do; as consequence the enzyme was inactivated after two cycles of reaction. This problem is the aim of numerous studies and we are working hard in this direction by coupling different catalytic systems for the *in situ* gradual supply of H_2_O_2_ [13,29,30].

## 3. Materials and Methods

### 3.1. Reagents and Enzymes

ABTS, hemoglobin from bovine blood, the *S. cerevisiae* transformation kit, the activated Thiol–Sepharose^®^ and fluoresecein isothiocyanate were all purchased from Sigma-Aldrich/Merck (Saint Louis, MO, USA). The uracil independent and ampicillin resistance shuttle vector pJRoC30 was obtained from the California Institute of Technology (CALTECH, Pasadena, CA, USA). The protease deficient BJ5465 strain of *S. cerevisiae* was obtained from LGCPromochem (Barcelona, Spain) and the Zymoclean Gel DNA Recovery kit was from Zymo Research (Irvine, CA, USA). The restriction enzymes BamHI and XhoI were purchased from New England Biolabs (Ipswich, MA, USA), and the high fidelity iProof polymerase was acquired from Bio-Rad (Hercules, CA, USA). The oligonucleotides were synthesized by Isogen Life Science (De Meern, The Netherlands), and NBD, naphthalene, styrene, 2,2’-dithiodipyridine and 1,4-dithiothreitol were purchased from Acros Organics (Waltham, MA, USA). The Sepabeads EC-EP203 was kindly provided by Resindion S.R.L. (Italy). All chemicals were reagent-grade purity.

### 3.2. Culture Media

Minimal drop-out liquid medium contained sterile yeast nitrogen base (100 mL, 6.7%), a sterile yeast synthetic drop-out medium supplement without uracil (100 mL, 19.2 g/L), sterile raffinose (100 mL, 20%), ddH_2_O (700 mL) and chloramphenicol (1 mL, 25 g/L). Minimal drop-out plates contained a sterile yeast nitrogen base (100 mL, 6.7%), a sterile yeast synthetic drop-out medium supplement without uracil (100 mL, 19.2 g/L), sterile glucose (100 mL, 20%), bacto agar (20 g), chloramphenicol (1 mL, 25 g/L) and ddH_2_O (to 1000 mL). The expression medium contained YP (720 mL), KH_2_PO_4_ [pH 6.0] buffer (67 mL, 1 M), sterile galactose (111 mL, 20%), sterile MgSO_4_ (22 mL, 0.1 M), sterile haemoglobin (2.75 mL, 20 g/L), absolute ethanol (31.6 mL), chloramphenicol (1 mL, 25 g/L) and ddH_2_O (to 1000 mL). For large scale cultures, the expression medium was supplemented with more sterile hemoglobin (0.3 g/L). YP medium contained yeast extract (10 g), peptone (20 g) and ddH_2_O (to 650 mL). The YPD solution contained yeast extract (10 g), peptone (20 g), sterile glucose (100 mL, 20%), chloramphenicol (1 mL, 25 g/L) and ddH_2_O (to 1000 mL).

### 3.3. Construction of the PaDa-I-Cys Variant

The PaDa-I variant was used as a template to introduce the S221C mutation by site-directed mutagenesis. Two high-fidelity polymerase chain reactions (PCR) were carried out in a final volume of 50 µL, containing: (i) DMSO (3%), the RMLN primer (5′-CCTCTATACTTTAACGTCAAGG-3′: 0.5 µM), the CYS-REV primer (5′-CATACGGCTGAATTGGAAAAAACACCGTGCAGCATCCATATCTAG-3′: 0.5 µM), dNTPs (1 mM, 0.25 mM each), iProof DNA polymerase (0.02 U/µL) and the template (0.2 ng/µL); or (ii) DMSO (3%), the CYS-DIR primer (5′-CTAGATATGGATGCTGCACGGTGTTTTTTCCAATTCAGCCGTATG-3′: 0.5 µM), the RMLC primer (5′-GGGAGGGCGTGAATGTAAGC-3′: 0.5 µM), dNTPs (1 mM, 0.25 mM each), iProof DNA polymerase (0.02 U/µL) and the template (0.2 ng/µL). The following PCR programs were used for each reaction: (i) 98 °C for 30 s (1 cycle), 98 °C for 10 s, 47 °C for 25 s, 72 °C for 35 s (28 cycles), and a final elongation at 72 °C for 10 min (1 cycle); (ii) 98 °C for 30 s (1 cycle), 98 °C for 10 s, 52 °C for 25 s, 72 °C for 20 s (28 cycles), and elongation at 72 °C for 10 min (1 cycle). The PCR products were loaded onto a preparative agarose gel and then purified using the Zymoclean Gel DNA Recovery kit. The pJRoC30 expression shuttle vector was used to clone the PCR fragments under the control of the GAL1 promoter. It was digested using BamHI and XhoI in order to obtain linearized plasmid and remove the parent gene. Then, it was loaded onto a preparative agarose gel and purified using the Zymoclean Gel DNA Recovery kit. The mutated PCR products were mixed with the linearized vector at a ratio of 2:1 (PCR product:linearized plasmid) and transformed into competent *S. cerevisiae* cells. Because of the length of the primers used, overlapping ~50 bp flanking regions were created for each segment to maximize the efficiency of in vivo DNA splicing between fragments by the yeast through IVOE [31].

### 3.4. DNA Sequencing

The plasmid containing the PaDa-I-Cys gene was sequenced using an ABI 3730 DNA Analyzer/Applied Biosystems Automatic Sequencer from Secugen.

### 3.5. Protein Modeling

The PaDa-I crystal structure at a resolution of 1.5 Å (Protein Data Bank Europe [PDB] accession number 5OXU) was used as a scaffold for protein modeling by PyMOL Molecular Graphics System, Version 2.0 Schrödinger, LLC.

### 3.6. Activation of Sepabeads with Disulfide Bonds

Sepabeads EC-EP203 were activated as indicated previously [23], with some modifications. Firstly, the content of available epoxy groups in the carrier was measured with a pH-stat by continuous titration with Na_2_S_2_SO_4_. The carrier (1 g) was mixed with Na_2_S_2_SO_4_ (600 mM) and titrated with HCl (1 N) until the reaction pH was 7.0. The carriers were incubated for 3 days at room temperature with Na_2_S_2_SO_4_ (600 mM) as this step was previously shown to improve the yield of the next reaction with 1,4-dithiothreitol (DTT). Subsequently, the carriers were washed with distilled water and incubated at room temperature for 24 h with DTT in EDTA (ethylenediaminetetraacetic acid, 1 mM) and sodium bicarbonate pH 8.5 (200 mM), at an epoxy:DTT ratio of 1:4. The carriers were then washed with sodium bicarbonate pH 8.5 (100 mM), distilled water, sodium acetate pH 5.0 (200 mM) and potassium phosphate pH 7.0 (50 mM). Finally, the SH groups were activated by addition of 2,2’-dithiodipyridine (2-PDS, 300 mM) in acetone:sodium bicarbonate (50 mM) 60:40 (*v*/*v*). After a 1 h incubation, the carriers were washed with acetone:water 60:40 (*v*/*v*), EDTA (1 mM) and potassium phosphate pH 7.0 (50 mM).

### 3.7. Directed Unique-Point Covalent Immobilization (DUCI)

Before immobilization, the PaDa-I-Cys mutant was incubated for 1 h in potassium phosphate buffer pH 7.0 containing DTT (25 mM), and the excess DTT was eliminated by gel chromatography using a pre-packed PD-10 column (GE-Healthcare, Chicago, IL, USA). Immobilization was performed by incubating PaDa-I-Cys (8 mL) with either carrier (C-I and C-II; 1 g wet weight each) for 72 h at room temperature. After immobilization, the biocatalyst was washed thoroughly with buffer in order to eliminate any non-specific binding. The immobilization yield was calculated as a percentage of the total units of PaDa-I-Cys bound to the carrier relative to the units added to the immobilization mixture, measured with ABTS as the reducing substrate. A negative control using PaDa-I was assessed following the same protocol.

### 3.8. Fluorescence Confocal Microscopy

To characterize the distribution of the enzyme in the carriers, fluorescence confocal microscopy was performed with fluorescein isothiocyanate (FITC) conjugated protein. First, the free cysteine of the UPO-S221C mutant was blocked to avoid FITC binding and then, the enzymatic extract was incubated with 2-PDS (300 mM) in acetone:sodium bicarbonate (50 mM) 60:40 (*v*/*v*) for 1 h. FITC dissolved in dimethylformamide was then added at a FITC/protein ratio of 5 µg mg^−1^ and the reaction was left for 1 h in sodium carbonate buffer pH 9.0 (50 mM). After labeling, the cysteine was again reduced by incubating 1 h with DTT (50 mM) in potassium phosphate buffer pH 7.0 (50 mM). The labeled protein was purified from the unbound FITC and DTT by gel chromatography using a pre-packed PD-10 column (GE-Healthcare). A negative control using PaDa-I subjected to the same protocol was used in all the assays. Fluorescence confocal microscopy was performed on a Leica TCS SP5 with AOBS (acousto-optical beam splitter), a tandem-scanning system SP5 II standard and a resonant scanner. A 20×/0.70 objective was used for all measurements. The laser produced FITC excitation at 488 nm and the fluorescent light emitted was detected at 520 nm.

### 3.9. Biochemical Characterization

The pH stability was measured by incubating the immobilized enzymes at 25 °C and 1000 rpm (Vortem Incubator) at different pH values in citrate-phosphate-borate buffer (200 mM). After a 1 h incubation, the residual activity was measured with ABTS (0.3 mM: ε_418_ = 36,000 M^−1^ cm^−1^) in sodium phosphate/citrate buffer pH 4.4 (100 mM) and H_2_O_2_ (2 mM). Thermostability was measured by incubating the immobilized enzymes at 1000 rpm (Vortem Incubator) in potassium phosphate buffer pH 7.0 (50 mM) at 35, 45, 55 and 65 °C. The value for 100 % of activity was taken as the initial activity of each enzyme without incubation (room temperature, 25 °C). The residual activity on ABTS was measured after a 1 h incubation. Activity in the presence of acetonitrile (ACN) was measured by adding increasing amounts of ACN to the reaction buffer with ABTS. All experiments were performed in triplicate using 20 mg of the immobilized enzyme in a final volume of 300 µL. The same measurements were obtained from the soluble enzymes (PaDa-I and PaDa-I-Cys in the same reaction mixtures and conditions (performed in triplicate).

### 3.10. Activity Colorimetric Assays

ABTS activity was measured as reported above, and the NBD assay (ε_425_= 9,700 M^−1^ cm^−1^) was performed in potassium phosphate buffer pH 7.0 (100 mM) containing NBD (1 mM), ACN (15%, *v*/*v*) and H_2_O_2_ (1 mM). Activities were measured in kinetic mode for the soluble enzymes and as the end-point after 1 min with the immobilized samples (measured in triplicate).

### 3.11. High-Performance Liquid Chromatography (HPLC) Analysis

The styrene epoxidation reaction took place in potassium phosphate buffer pH 7.0 (10 mM) containing styrene (1 mM), methanol (5%) and H_2_O_2_ (1 mM)_._ Naphthalene hydroxylation was performed in potassium phosphate buffer pH 7.0 (100 mM) containing naphthalene (0.5 mM), ACN (10%) and H_2_O_2_ (1 mM), and samples were taken at different times and analyzed by HPLC. Chromatography was performed with a 9012 pump (Varian) and an ACE3 C18-PFP column (150 × 4.6 mm, ACE) maintained at 40 °C, detecting the products with a photodiode array (PDA) at 297 nm for the naphthalene reaction and at 220 nm for the styrene reaction. Methanol:water 85:15 (*v*/*v*) was used as the mobile phase for the naphthalene reaction and methanol:water 75:25 (*v*/*v*) for the styrene reaction, at a flow rate of 1 mL/min in both cases. In order to quantify the reactions, calibration curves of 1-naphthol and styrene oxide were obtained and integrated using Varian Star 4.0 Software.

## 4. Conclusions

UPO is one of the most versatile biocatalysts yet reported for oxyfunctionalization chemistry. Having recently resolved the crystal structure of the evolved PaDa-I, and given the availability of a well-established laboratory evolution platform and a robust tandem-yeast expression system, the enzyme immobilization procedure described here fills the gap for its future commercial implementation. The proof of concept for UPO immobilization by DUCI presented here could be easily translated to other well-known ligninolytic evolved systems (e.g., laccases, versatile peroxidases), opening a range of opportunities in distinct industrial areas with particular emphasis on pharmaceuticals, fine chemistry, building blocks or lignocellulose biorefineries.

## 5. Patents

Rodríguez Buey, M.; García Ruiz, M.; Martín Díaz, J.; Santos Moriano, P.C.; Molina Espeja, P.; García Ruiz, E.; Plou Gasca, F.J.; Alcalde Galeote, M. Mutants of unspecific peroxygenase with high monooxygenase activity and uses thereof. EP17382151.3

## Figures and Tables

**Table 1:** Peroxygenative:peroxidative activity ratio for PaDa-I and PaDa-I-Cys.

	PaDa-I	PaDa-I-Cys
NBD/ABTS ^1^	0.14	0.16
Total ABTS U in culture	1673	1019

^1^ Activity for NBD (5-nitro-1,3-benzodioxole) was measured in potassium phosphate buffer (pH 7.0, 100 mM) containing NBD (1 mM), acetonitrile (ACN) (15%, *v*/*v*) and H_2_O_2_ (1 mM). Activity for ABTS (2,2′-azino-bis(3-ethylbenzothiazoline-6-sulfonic acid)) was measured in sodium phosphate/citrate buffer (pH 4.4, 100 mM) containing ABTS (0.3 mM) and H_2_O_2_ (2 mM).

## Abbreviations

2-PDS	2,2’-dithiodipyridine
ABTS	2,2′-Azino-bis(3-ethylbenzothiazoline-6-sulfonic acid)
ACN	Acetonitrile
AOBS	Acousto optical beam splitter
C-I	Sepabeads EC-EP203
C-II	Thiol-Sepharose^®^
DTT	1,4-Dithiothreitol
DUCI	Directed unique-point covalent immobilization
EDTA	Ethylenediaminetetraacetic acid
FITC	Fluorescein isothiocyanate
IVOE	In vivo overlap extension
NBD	5-Nitro-1,3-benzodioxole
PaDa-I	Evolved mutant from the wild type UPO secreted by *Agrocybe aegerita*
PaDa-I-Cys	PaDa-I with the S221C mutation for DUCI immobilization
PDB	Protein data bank
UPO	Unspecific peroxygenase
